# SPR741, an Antibiotic Adjuvant, Potentiates the *In Vitro* and *In Vivo* Activity of Rifampin against Clinically Relevant Extensively Drug-Resistant Acinetobacter baumannii

**DOI:** 10.1128/AAC.01239-17

**Published:** 2017-11-22

**Authors:** Daniel V. Zurawski, Alexandria A. Reinhart, Yonas A. Alamneh, Michael J. Pucci, Yuanzheng Si, Rania Abu-Taleb, Jonathan P. Shearer, Samandra T. Demons, Stuart D. Tyner, Troy Lister

**Affiliations:** a Wound Infections Department, Bacterial Diseases Branch, Walter Reed Army Institute of Research, Silver Spring, Maryland, USA; b Spero Therapeutics, Inc., Cambridge, Massachusetts, USA

**Keywords:** Acinetobacter, ESKAPE pathogens, animal models, antibacterial, antibiotic adjuvants, antibiotic resistance, antibiotics, mouse model, pulmonary model, virulent strain

## Abstract

Acinetobacter baumannii is responsible for 10% of all nosocomial infections and has >50% mortality rates when causing ventilator-associated pneumonia. In this proof-of-concept study, we evaluated SPR741, an antibiotic adjuvant that permeabilizes the Gram-negative membrane, in combination with rifampin against AB5075, an extensively drug-resistant (XDR) A. baumannii strain. In standard *in vitro* assays and in a murine pulmonary model, we found that this drug combination can significantly reduce bacterial burden and promote animal survival despite an aggressive infection.

## TEXT

Acinetobacter baumannii gained notoriety as the bacterial species most frequently isolated from U.S. soldiers from 2004 to 2010, with >3,500 infections associated with war wounds (1–4). Presently, A. baumannii is a significant problem worldwide because of extensively drug-resistant (XDR) strains and the aggressive nature of some infections causing ventilator-associated pneumonia (5–7). Recently, the World Health Organization cited a critical need for A. baumannii research because of increased drug resistance and lack of treatments (8). To address the immediate need, research strategies such as antibiotic adjuvants, which involve the current antibiotic armamentarium, may provide a faster path to clinical application.

Antibiotic adjuvants are typically small molecules that sensitize a bacterium to clinically approved antibiotics (9). Specific examples with respect to A. baumannii include 2-aminoimidazole-based compounds, which disrupt two-component signaling (10), and anthracyclines, which potentiate activity of rifampin and linezolid (11). Here, we evaluated SPR741 (formerly NAB741), a polymyxin-B-derived molecule specifically designed to minimize the nephrotoxicity associated with this antibacterial class (12). SPR741 has reduced positive charge and lacks the highly lipophilic fatty-acid side chain present in polymyxins, which are the two structural features responsible for clinical nephrotoxicity (12, 13). As a proof of concept, we tested a combination of SPR741 and rifampin because colistin and rifampin have proven to be an effective combination in both mouse models and in patients (14–18). Rifampin was also previously tested against AB5075, a highly virulent XDR strain, in a murine pulmonary model of infection (19), which facilitated dosing for this study.

**FIG 1:**
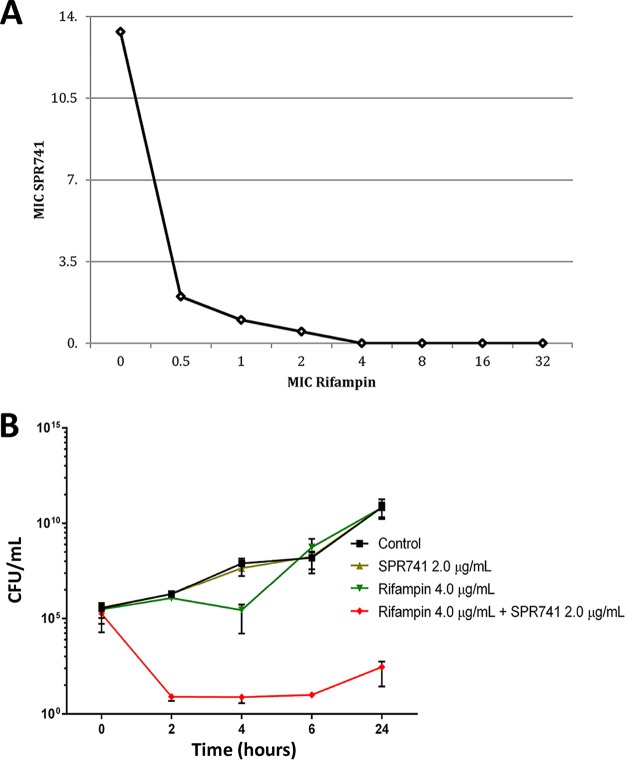
(A) An isobologram generated from checkerboard assays where AB5075 was grown with increasing concentrations of SPR741 and rifampin. (B) Time-kill assay of SPR741, rifampin, and combinations against XDR-A. baumannii. AB5075 was grown overnight in CAMHB, then subcultured into CAMHB for 2 h. Cultures were inoculated 1:10 into CAMHB alone or with 2.0 μg/ml SPR741, 1.0 μg/ml rifampin, or both SPR741 and rifampin at their respective concentrations. Time points were taken at 0, 2, 4, 6, and 24 h, and samples were plated for CFU. Only the combination of rifampin and SPR741 was statistically significant (red line), as tested by two-way ANOVA (*P* = 0.0048).

First, the SPR741/rifampin combination was tested *in vitro* against A. baumannii. The MICs for SPR741 and rifampin were determined for AB5075 to be 128 μg/ml and 4.0 μg/ml, respectively, using standard CLSI methods in cation-adjusted Mueller-Hinton broth (CAMHB) (20). This combination was assessed with the checkerboard method to determine fractional inhibitory concentration (FIC) (21), where synergistic activity was defined by an FIC of ≤0.5 (22). The MIC of rifampin dropped from 4.0 to 0.5 μg/ml in the presence of 2.0 μg/ml SPR741, an 8-fold reduction, thus producing an FIC of 0.14 and indicating synergy. An isobologram was generated from these results (Fig. 1A).

Next, we examined whether this synergy was applicable across the whole species of A. baumannii. We analyzed a previously described 28-strain diversity set (19) and determined MICs for all strains. A combination of 4.0 μg/ml of SPR741 and 1.0 μg/ml rifampin inhibited the growth of 96% of the strains, with a minimum 4-fold reduction of most MICs. AB3027 was the exception, as it is significantly resistant to rifampin (MIC > 128 μg/ml; Table 1).

**TABLE 1:** A. baumannii strains used in this study with individual MIC values

A. baumannii strain	MIC for^*a*^:		Growth in presence of SPR741 (4.0 μg/ml) + rifampin (1.0 μg/ml)^*b*^
	Rifampin	SPR741	
AB967	4	<64	−
AB2828	2	256	−
AB3340	2	256	−
AB3560	4	128	−
AB3638	2	256	−
AB3785	4	128	−
AB3806	2	256	−
AB3927	>256	256	+
AB4025	4	128	−
AB4026	4	>256	−
AB4027	4	>256	−
AB4052	4	256	−
AB4269	8	>256	−
AB4448	4	<64	−
AB4456	4	128	−
AB4490	4	128	−
AB4498	4	256	−
AB4795	2	128	−
AB4857	4	256	−
AB4878	4	256	−
AB4932	16	<64	−
AB4957	4	256	−
AB4991	4	128	−
AB5001	4	256	−
AB5075	2	128	−
AB5197	4	256	−
AB5256	4	128	−
AB5674	2	128	−
AB5711	4	128	−

a MICs were determined separately in the presence of the combination of SPR741 at 4.0 μg/ml and rifampin at 1.0 μg/ml.

b −, no growth; +, growth.

To further evaluate activity, time-kill assays (3 biological replicates) were performed with 2.0 μg/ml SPR741 and 1.0 μg/ml rifampin against AB5075 grown in CAHMB as previously described (23). Each drug used alone had little effect on growth. In contrast, the combination resulted in fewer than 10 organisms (limit of detection) on LB plates at 2, 4, and 6 h (Fig. 1B), a significant result (two-way ANOVA, *P* = 0.0048). This result confirmed that the combination of SPR741/rifampin had a bactericidal, synergistic effect that should be further tested *in vivo*.

**FIG 2:**
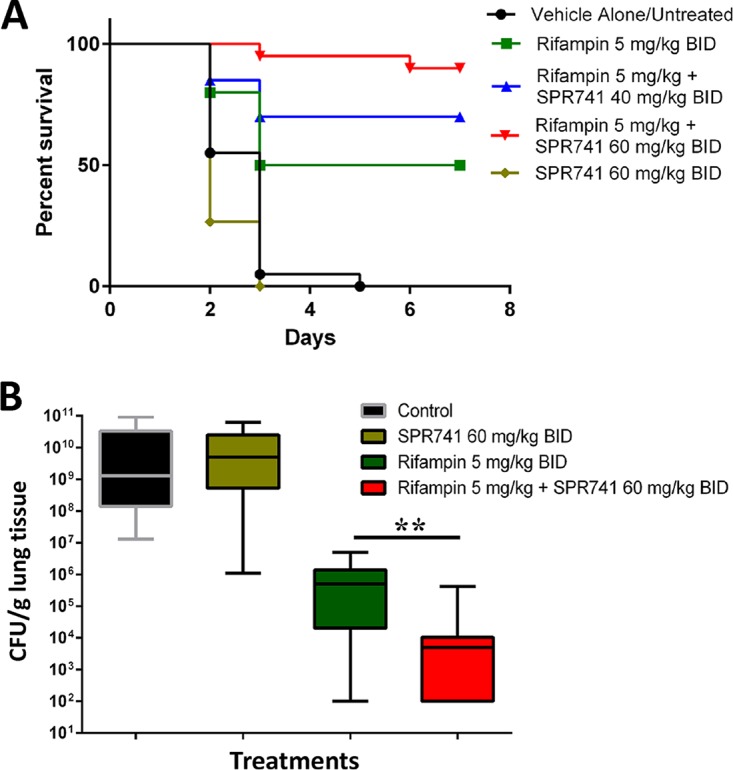
(A) Mice were intranasally inoculated with 5.0 × 10^6^ CFU AB5075 and treated with rifampin 5 mg/kg BID (green line), SPR741 60 mg/kg BID (yellow line), the combination of these doses at 40 mg/kg SPR741 BID (blue line) or 60 mg/kg SPR741 BID (red line), or sterile saline (vehicle alone, untreated control; black line). The data presented is a combination of two biological replicates of 10 mice/group for a total of 20 mice (*n* = 20). Mice were monitored daily for signs of morbidity and mortality. Results for all groups were statistically significant (*P* < 0.05) compared to each other via the Mantel-Cox test (Graphpad Prism), except for the untreated control (black line) and SPR741-alone groups (yellow line). (B) Box-and-whisker plots of log_10_ CFU/g of lung tissue on day 2 postinoculum. Mice were treated with rifampin at 5 mg/kg BID (green box), SPR741 60 mg/kg BID (yellow box), the combination of these doses (red box), or sterile saline (vehicle alone, control; black box) for 2 days. Boxes show median and interquartile ranges, while whiskers represent 95% confidence interval (CI). Groups were compared each day via the Mann-Whitney U test. ** represents *P* values of <0.01 (*P* = 0.0029). These data are pooled from two biological replicates with at least 6 mice per group and 13 to 16 mice total per test condition.

The methods for the murine pulmonary model of A. baumannii infection were previously detailed (19), and were conducted similarly for this study. In pilot experiments, we initially evaluated three doses of SPR741 (40, 60, or 80 mg/kg) once daily (QD) or twice daily (BID), with 5.0 mg/kg or 10.0 mg/kg doses of rifampin also provided QD or BID. Only the BID-treated mice survived over the course of 1 week, suggesting that QD treatment was not sufficient (data not shown). Next, two independent experiments were conducted using 10 mice per group. Four hours after A. baumannii inoculation, groups were treated with sterile saline (negative control), 5.0 mg/kg rifampin, 60 mg/kg SPR741 BID, or the combination of SPR741 40 mg/kg or 60 mg/kg SPR741 with 5.0 mg/kg rifampin BID for the next 3 days. The survival rate for 60 mg/kg SPR741 combined with 5.0 mg/kg rifampin BID was 90% (Fig. 2A), a significant success for this aggressive infection model (Mantel-Cox test, *P* < 0.0027). In contrast, untreated animals or mice receiving SPR741 alone succumbed to infection (Fig. 2A). Rifampin alone only provided 50% survival (Fig. 2A).

Separate experiments were then conducted to evaluate bacterial burden via CFU (CFU/g of lung tissue). Mice were sacrificed on day 2 before the untreated control animals succumbed to infection, as previously described (19). These results mirrored the survival results, where the combination of SPR741/rifampin decreased bacterial burden by 6.0 log_10_ CFU/g compared to the vehicle-alone control (Mann-Whitney U test, *P* < 0.0001) (Fig. 2B). When comparing the combination of SPR741/rifampin to rifampin treatment alone, a 2.0-log_10_ reduction in burden was seen with the addition of SPR741, which was also statistically significant (Mann-Whitney U test, *P* = 0.0029) (Fig. 2B).

This investigation is a promising start with regard to *in vivo* safety and efficacy of SPR741 combinations against Gram-negative pathogens. In pilot experiments, more mice did succumb (80% survival) with 80 mg/kg BID doses of SPR741. The reason for this is unclear, but clearance and complete animal survival are difficult to achieve in this model. AB5075 is highly aggressive and bacteria reach high numbers in lung tissue, followed by dissemination into the bloodstream and colonization of other organs, including heart, spleen, and kidneys (4, 19). With regard to toxicity, previously presented results (P. Shastri and S. Coleman, ASM Microbe, Boston, MA, 16 to 20 June 2016) determined that the 60 mg/kg dose in mice scales to a human dose of approximately 200 to 400 mg. SPR741 demonstrated a no-observed-adverse-effect-level (NOAEL) of >60 mg/kg/day in cynomolgus monkeys (S. Coleman, M. Bleavins, T. Lister, M. Vaara, and T.R. Parr, ASM Microbe, Boston, MA, 16 to 20 June 2016), while nephrotoxicity was observed at 12 mg/kg/day with polymyxin B. Spero Therapeutics recently completed SPR741 dosing in healthy volunteers (https://clinicaltrials.gov/ct2/show/NCT03022175, ClinicalTrials registration no. NCT03022175). With these prior results and the data obtained from this proof-of-concept study, more preclinical investigations of SPR741-antibiotic combinations are warranted to evaluate efficacy against other bacterial species. Furthermore, animal models mimicking other clinical indications and evaluating pharmacokinetics/pharmacodynamics (PK/PD) are also currently being pursued.
